# Association between perceived weight discrimination and physical activity: a population-based study among English middle-aged and older adults

**DOI:** 10.1136/bmjopen-2016-014592

**Published:** 2017-03-06

**Authors:** Sarah E Jackson, Andrew Steptoe

**Affiliations:** Department of Epidemiology and Public Health, University College London, London, UK

**Keywords:** weight-related discrimination, stigma, physical activity, population studies

## Abstract

**Objective:**

To examine the association between perceived weight discrimination and physical activity in a large population-based sample.

**Design:**

Data were from 2423 men and 3057 women aged ≥50 years participating in Wave 5 (2010/11) of the English Longitudinal Study of Ageing. Participants reported experiences of weight discrimination in everyday life and frequency of light, moderate and vigorous physical activities. We used logistic regression to test associations between perceived weight discrimination and physical activity, controlling for age, sex, socioeconomic status and body mass index (BMI).

**Results:**

Perceived weight discrimination was associated with almost 60% higher odds of being inactive (OR 1.59, 95% CI 1.05 to 2.40, p=.028) and 30% lower odds of engaging in moderate or vigorous activity at least once a week (OR 0.70, 95% CI 0.53 to 0.94, p=.017).

**Conclusions:**

Independent of BMI, individuals who perceive unfair treatment on the basis of their weight are less physically active than those who do not perceive discrimination. This has important implications for the health and well-being of individuals who experience weight-based discrimination, and may also contribute to a cycle of weight gain and further mistreatment.

**Strengths and limitations of this study**

- First study to examine the relationship between weight discrimination and physical activity in a large population sample;
- use of a large, nationally representative sample provides robust estimates of observed associations;
- discrimination and physical activity were assessed alongside a vast number of other measures, minimising reporting bias, but both were self-reported global assessments which may be limited by inaccuracies in reporting;
- height and weight were objectively measured, providing an accurate measure of body mass index, although these data were collected 2 years prior to the discrimination data and changes in weight may have occurred in the interim;
- cross-sectional study design prohibits causal inferences.

## Introduction

Recent decades have seen concurrent rises in obesity prevalence and stigmatisation of, and discrimination against, people who carry excess weight.^1^ National surveys in the UK and the USA have revealed reports of weight-related mistreatment by up to 7%, 14% and 43% of individuals with a body mass index (BMI) in the overweight, moderately obese and severely obese ranges, respectively.^1^ ^2^

In addition to well-documented harmful consequences for emotional well-being,^3^ ^4^ there is increasing evidence that weight discrimination may adversely affect weight-related behaviours, for example, increasing intake of high-fat and high-calorie foods,^5^ decreasing dietary quality^6^ and limiting physical activity,^7–11^ although findings have been somewhat contradictory. Studies have shown that children, adolescents and adults exposed to weight stigma are more likely to avoid physical activity, even after adjustment for BMI.^7^ ^8^ ^10^ ^12^ However, associations between experiences of weight stigma and actual exercise behaviour have not been consistently observed. An experimental study in children observed decreased participation in physical activity following simulated ostracism.^11^ In two small adult samples (n=100–111), experiences of weight stigma were not associated with self-reported mild, moderate or strenuous physical activity, despite significant negative correlations between stigma experiences and reported avoidance of physical activity and between avoidance of physical activity and self-reported exercise behaviour.^7^ ^8^ In an online sample of 177 women, individuals who had experienced weight stigma reported *higher* levels of physical activity, despite being less likely to report believing that weight was under personal control.^13^

This study aimed to help resolve these discrepant findings by using data from a large (n>5000) nationally representative sample taking part in the English Longitudinal Study of Ageing (ELSA) to examine the association between perceived weight discrimination and self-reported physical activity.

## Method

### Study population

ELSA is a longitudinal panel study of men and women aged ≥50 years living in England that started in 2002. Participants are assessed on a two-yearly basis, with a nurse visiting the home to obtain objective measurements of health status, including weight in alternate (even) waves. Wave 5 (2010/11) included items assessing discrimination. Of the 9090 participants interviewed in Wave 5, 7574 completed the questionnaire that measured experiences of discrimination and provided data on physical activity. The present study uses these data as well as BMI measurements collected in Wave 4 (2008/09), as no anthropometric measurements were taken in Wave 5. Our final analytic sample comprised 5480 participants for whom complete data were available.

### Measures

Perceived weight discrimination was assessed using items based on those developed and used widely in US longitudinal studies.^1^ ^14^ Participants were asked how frequently they experience five discriminatory situations in their day-to-day life: ‘(1) you are treated with less respect/courtesy; (2) you receive poorer service than other people in restaurants/stores; (3) people act as if they think you are not clever; (4) you are threatened/harassed and (5) you receive poorer service or treatment than other people from doctors/hospitals,’ with responses on a scale from ‘never’ to ‘almost every day’. The majority of participants reported never experiencing discrimination, skewing the distribution of the data, so responses were dichotomised to distinguish between these respondents and those who had ever experienced discrimination in any domain (never vs all other options). Individuals reporting discrimination in at least one domain were asked to indicate the reason(s) they attributed their experience to from a list of characteristics including weight, age, sex, race, physical disability, an aspect of physical appearance, sexual orientation, financial status or other reason. Those who attributed any experience of discrimination to their weight are treated as cases of perceived weight discrimination for our analyses.

Physical activity was self-reported in response to three questions on the frequency of participation in light, moderate and vigorous activities (more than once a week/once a week/one to three times a month/hardly ever or never). For the present analyses, physical activity was further categorised into four groups: inactive (no activity on a weekly basis); only light activity at least once a week; at least moderate but no vigorous activity at least once a week and any vigorous activity at least once a week. These thresholds were selected based on previous work in ELSA demonstrating robust dose–response associations with mortality.^15^ The main outcomes of interest were inactivity and moderate or vigorous activity at least once a week.

Age, sex, household non-pension wealth (a sensitive measure of socioeconomic status (SES) in this age group) and BMI (from measured height and weight) were included as covariates.

### Statistical analysis

We used weights to correct for sampling probabilities and differential non-response and to calibrate back to the 2011 national census population distributions for age and sex. We used adjusted percentages to compare the distribution of participants who did and did not report weight discrimination across physical activity classifications, controlling for age, sex, SES and BMI. We analysed associations between perceived weight discrimination and physical activity using logistic regression, with age, sex, SES and BMI as covariates and the no weight discrimination group as the reference category. Interactions between weight discrimination and BMI were tested in order to explore whether associations between weight discrimination and engagement in physical activity differed according to the degree of overweight.

## Results

Perceived weight discrimination was reported by 4.9% of participants. Weight discrimination varied substantially by weight status (p<0.001), rising from 0.8% in underweight and normal weight participants (n=1451) and 0.9% in overweight participants (n=2272) to 13.4% in individuals with obesity (n=1757). Those who reported weight discrimination tended to be younger and less wealthy, but the groups did not differ significantly by sex (table 1).

**Table 1:** Sample descriptive characteristics—mean (SD) or % (n)

	No weight discrimination (n=5212)	Weight discrimination (n=268)	p Value
Age, years	67.71 (8.95)	62.54 (6.89)	<0.001
Sex			
Male	44.5 (2319)	38.8 (104)	0.067
Female	55.5 (2893)	61.2 (164)	–
Wealth quintile			
1 (poorest)	15.3 (799)	26.1 (70)	<0.001
2	19.0 (992)	28.4 (76)	–
3	19.9 (1039)	17.2 (46)	–
4	22.3 (1164)	15.7 (42)	–
5 (richest)	23.4 (1218)	12.7 (34)	–
BMI, kg/m^2^	27.93 (4.89)	36.49 (6.69)	<0.001

Unweighted data.

BMI, body mass index.

**Figure 1:**
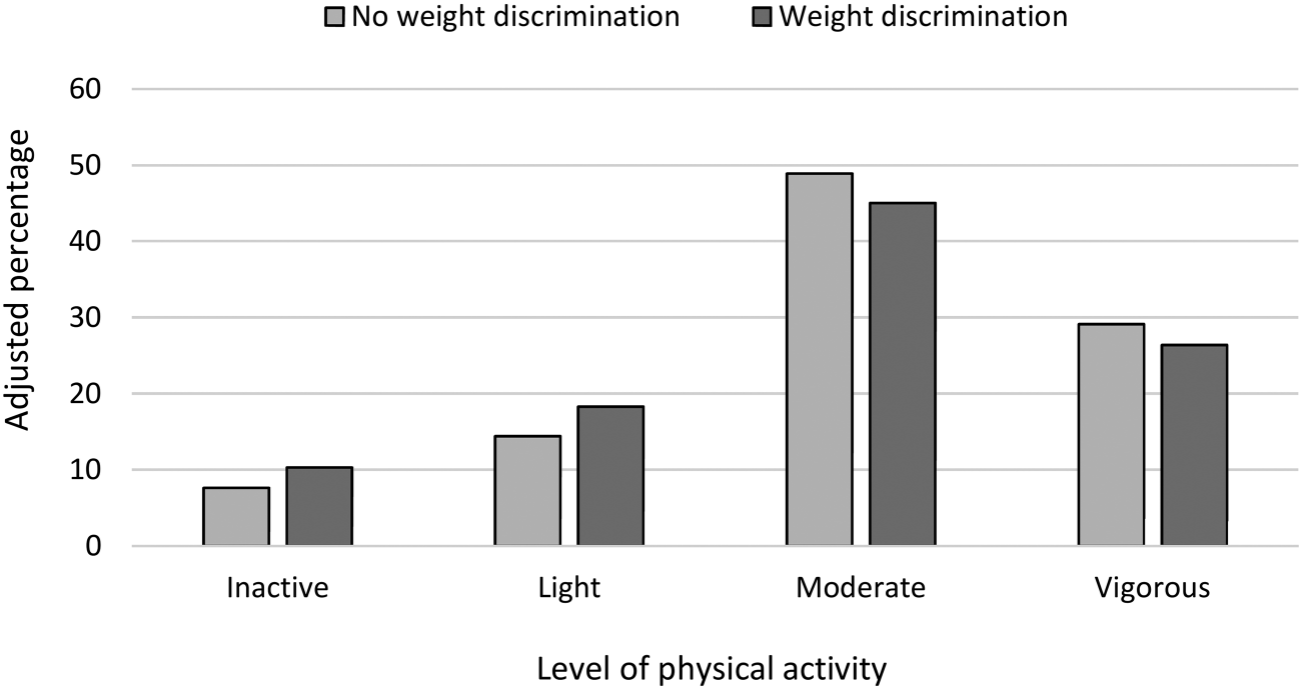
Level of physical activity in individuals reporting experiences of weight discrimination (n=268) and individuals reporting no weight discrimination (n=5212) in the English Longitudinal Study of Ageing, adjusted for age, sex, SES and BMI. BMI, body mass index; SES, socioeconomic status.

Figure 1 presents data on activity level by perceived weight discrimination. Among participants who reported weight discrimination, 10.3% reported no regular physical activity, 18.3% reported only light activity at least once a week and moderate and vigorous intensity activity was reported in 45.0% and 26.4%, respectively. Rates of inactivity and light activity were comparatively lower in the group which did not report weight discrimination, at 7.6% and 14.4% respectively, and rates of moderate and vigorous activity were higher, at 48.9% and 29.1% respectively. Logistic regression models confirmed these differences, indicating that perceived weight discrimination was significantly associated with almost 60% higher odds of being inactive (OR 1.59, 95% CI 1.05 to 2.40, p=.028) and 30% lower odds of engaging in regular moderate or vigorous activity (OR 0.70, 95% CI 0.53 to 0.94, p=.017). Interactions between weight discrimination and BMI were not significant for either outcome.

## Discussion

This study is the first to examine the relationship between weight discrimination and physical activity in a large population-based sample. Previous studies have shown increased motivation to avoid exercise in people exposed to weight stigma.^7^ ^8^ ^10^ ^12^ Our results are consistent with these findings, demonstrating an association between self-reported experiences of weight discrimination and engagement in physical activity in a sample of middle-aged and older adults. Independent of differences in physical activity related to BMI, we saw that participants who reported weight-based discrimination were substantially more likely to report being sedentary, and less likely to report regularly engaging in moderately or vigorously energetic activities.

Previous studies that have examined associations between weight stigma and actual exercise behaviour have produced mixed results.^7^ ^8^ ^11^ ^13^ Our finding linking weight discrimination with reduced physical activity contrasts with one previous study in particular that observed increased levels of physical activity among women who had experienced weight stigma,^13^ but is consistent with two other key findings from the same study. First, the results showed that participants with experience of weight stigma were less likely to believe weight was controllable, which might reduce motivation to engage in exercise for weight control purposes. Second, weight bias internalisation (self-directed stigma) was associated with lower levels of physical activity.

There are several reasons weight discrimination might be related to lower levels of physical activity. Avoidance of domains in which discrimination is likely to occur is a common response to social identity threat.^16^ Individuals who perceive discrimination may be more self-conscious about exercising in front of people for fear that it might attract undesirable attention. In a population survey, more than one in five adults with obesity reported being embarrassed and feeling too fat to exercise,^17^ and studies in children indicate reluctance to participate in school-based physical activity because of concerns over potential weight-related teasing.^18^ Internalisation of weight bias may also result in a loss of self-efficacy and motivation to achieve goals, leaving people wondering why they should bother trying to be active.^13^

The observed association with lower physical activity has implications for the health and well-being of those who experience weight-based discrimination. Regular physical activity has benefits for primary and secondary prevention of numerous chronic diseases and reduces the risk of premature death.^19^ Given that individuals with obesity are already at increased risk for developing many of these conditions,^20^ it is particularly important to promote physical activity in this group. Importantly in the context of weight discrimination, lower engagement in physical activity may lead to increased obesity (even among the already obese) and thus to increased risk of further discrimination. Physical activity may be an important mediator of previously demonstrated associations between weight discrimination, weight gain and persistent obesity.^14^ ^21^

The relationship between weight discrimination and physical activity did not differ significantly by BMI, indicating that individuals who experience weight discrimination are likely to be less physically active, regardless of their weight. However, it should be noted that very few (<1%) participants with a BMI <30 reported weight discrimination, so this analysis may have been underpowered. It is possible that a person who encounters weight discrimination may lose weight but still avoid exercise. Given the substantial health benefits of being physically active, interventions that aim to reduce weight bias at a population level—for example, through schools, local communities or national campaigns—may have a greater impact on health than those that encourage people to lose weight. A Health at Every Size approach may be helpful in promoting the adoption of healthy habits, including regular physical activity, for the sake of health and well-being as opposed to weight control.^22^ There has been some evidence indicating long-term beneficial effects of such an approach on eating behaviours.^23^

This study had limitations. Physical activity data were self-reported, but a previous comparison in an ELSA subsample showed a moderate correlation with objective assessments (Spearman's *r*=0.21, p=.020).^24^ Based on the data in ELSA, we were unable to define the nationally recommended physical activity threshold (2.5h of moderate/vigorous physical activity weekly). However, previous research supports our chosen threshold as a meaningful predictor of health outcomes in this cohort.^15^ It was not possible to analyse variations in the intensity of discrimination, which may be differentially associated with physical activity. Weight discrimination was determined by self-reports of past experiences and as a result may have been underreported due to memory or recall biases. Participants could attribute experiences of discrimination to one or more personal characteristics from a presented list; although this did offer advantages with regard to blinding the present study's focus on weight-related discrimination and thereby potentially reducing bias among heavier respondents. Items measuring discrimination covered five generic domains (eg, being treated with less respect or receiving poorer service in restaurants/stores), but did not assess experiences that may be specific to weight discrimination (eg, being required to pay for two passenger seats when travelling by plane) and their omission may again mean that the prevalence of weight discrimination was underestimated. The study was cross-sectional which prohibits inferences on causality, but previous research showing individuals exposed to weight stigma subsequently report an increased desire to avoid exercise is suggestive of a causal association.^8^ The timing of discriminatory experiences could not be ascertained from the questions used to assess discrimination, so it was not possible to control for prior physical activity levels. Height and weight were not measured at the same time as discrimination, but previous work in this cohort has shown very high stability in BMI over time,^25^ so it is unlikely that this greatly influenced the present results. Participants were from an older population, in which levels of physical activity are likely to be lower^26^ and experiences of weight discrimination are typically less common^1^ relative to younger populations so findings cannot be assumed to generalise. The prevalence of weight discrimination was lower in our sample than has been observed in previous studies that have examined wider age ranges,^1^ ^27^ but was very similar to rates of weight discrimination reported in comparable age groups in a large US sample, where rates were 5.3% in 55–64-year-olds and 4.0% in 65–74-year-olds.^27^

In summary, these results provide evidence that weight discrimination may be associated with lower participation in regular physical activity and higher rates of sedentary behaviour. Through this mechanism, weight discrimination may be implicated in the perpetuation of weight gain, onset of obesity related comorbidities and even premature mortality.
